# Ultrastructural study of the duck brain infected with duck Tembusu virus

**DOI:** 10.3389/fmicb.2023.1086828

**Published:** 2023-02-20

**Authors:** Sheng Yang, Yonghong Shi, Jingxian Wu, Qiusheng Chen

**Affiliations:** ^1^MOE Joint International Research Laboratory of Animal Health and Food Safety, College of Veterinary Medicine, Nanjing Agricultural University, Nanjing, Jiangsu Province, China; ^2^Shanghai Veterinary Research Institute, Chinese Academy of Agricultural Sciences, Shanghai, China

**Keywords:** duck tembusu virus, neuron, neuroglial cell, brain microvascular, ultrastructural study

## Abstract

Duck Tembusu virus (DTMUV) is an emerging avian flavivirus characterized by causing severe ovaritis and neurological symptoms in ducks. The pathology of the central nervous system (CNS) caused by DTMUV is rarely studied. This study aimed to systematically investigate the ultrastructural pathology of the CNS of ducklings and adult ducks infected with DTMUV *via* transmission electron microscopy technology at a cytopathological level. The results showed that DTMUV caused extensive lesions in the brain parenchyma of ducklings and slight damage in adult ducks. The neuron was the target cell of DTMUV, and virions were mainly observed in their cisternae of rough endoplasmic reticulum and the saccules of Golgi apparatus. The neuron perikaryon showed degenerative changes where the membranous organelles gradually decomposed and disappeared with DTMUV infection. Besides neurons, DTMUV infection induced marked swelling in astrocytic foot processes in ducklings and evident myelin lesions in ducklings and adult ducks. The activated microglia were observed phagocytizing injured neurons, neuroglia cells, nerve fibers, and capillaries after the DTMUV infection. Affected brain microvascular endothelial cells were surrounded by edema and had increased pinocytotic vesicles and cytoplasmic lesions. In conclusion, the above results systematically describe the subcellular morphological changes of the CNS after DTMUV infection, providing an ultrastructural pathological research basis for DTMUV-induced neuropathy.

## 1. Introduction

Duck Tembusu virus (DTMUV) attracted little public attention when it was first isolated from the Culex mosquito in Malaysia in 1955. DTMUV broke out in southeastern China and then quickly spread throughout Asia in 2010, resulting in huge economic losses to the poultry industry, especially the duck industry. DTMUV was the first flavivirus that caused acute, severe infection in duck flocks, characterized by reduced egg production in laying ducks and severe neurological syndrome in ducklings (Su et al., 2011). In addition, DTMUV has the potential to spread across species (Li et al., 2013; Tang et al., 2013). DTMUV antibodies and viral nucleic acids were discovered in duck factory workers (Tang et al., 2013). DTMUV could also infect mice and lead to severe nonsuppurative encephalitis (Li et al., 2013; Ti et al., 2016). Hence, it is necessary to study the potential pathogenicity of DTMUV in animals and humans.

The retention time of DTMUV in the brain is almost the longest (Li et al., 2013), and the nerve damage caused by virus infection is one of the host fatal causes (Luethy et al., 2016). In previous studies, DTMUV infection caused marked neurological symptoms in commercial meat ducks (Su et al., 2011). The neural tissue is mainly composed of neurons and neuroglial cells, of which the number of glial cells is about 10 to 50 times that of neurons (Kettenmann, 1999). Neurons are responsible for transmitting signals. Neuroglial cells support, nourish, insulate, and protect the neurons. Although the two types of cells in the same tissue differ in form and function, they are closely related, adapt to each other, and exert an overall function through interaction. The neurons are the main target cells of flavivirus, but they can also affect other cells in the brain. The pathological mechanism of DTMUV-infected neuron cells was reported minimally. As a new pathogen, DTMUV-induced duck encephalitis lacks a clear systematic pathological study.

Age is inversely related to susceptibility. It has been reported that the pathogenicity of flavivirus in mice concerns age (Salimi et al., 2016). Similarly, in avians, the cherry valley duck’s susceptibility to the virus reduced gradually along with age growth. When ducks under 2 weeks were more likely to be infected with viruses and had more obvious and severe clinical symptoms, while ducks more than 5 weeks had a lower susceptibility, morbidity, and mortality (Sun et al., 2014). Ducklings were more susceptible to West Nile virus (WNV) than adults (Shirafuji et al., 2009). Therefore, in this study, ducklings (7-day-old) and adult ducks (180 days old) were used to construct a neuro-symptomatic model. Our previous studies have demonstrated that DTMUV could replicate efficiently in the duckling brain and cause severe non-pyogenic encephalitis in ducklings. Virus content in the brain of ducklings infected with DTMUV peaked at 7–9 days post-infection (dpi) and then decreased. No obvious symptoms were observed in the duckling brain during the early stage of DTMUV infection (3 dpi). At 5 dpi, the ducklings began to show lethargy and loss of appetite, and at 7–12 dpi, the ducklings showed nervous symptoms such as necking and unstable standing (Yang et al., 2021). In the present study, we further systematically observed the ultrastructural changes at the subcellular (organelles) level of the duck brain after DTMUV infection, to elucidate the pathological mechanism of DTMUV-induced non-suppurative encephalitis in duck.

## 2. Materials and methods

### 2.1. Establishment of animal models and samples collections

Ducklings and adult ducks were purchased from Nanjing Qizai Biological Co., Ltd. (Nanjing, China). DTMUV purified strain XZ-2012 was provided by the College of Veterinary Medicine, Nanjing Agricultural University. The titer of the virus used in this experiment was 5 × 10^5^ TCID_50_/ml. A total of 160 7-day-old Shaoxing Muscovy ducklings were randomly divided into an experimental group and a control group. The ducklings in the experimental group were injected with 100 μl DTMUV solution through the jugular vein. The ducklings in the control group animals were given the same dose of phosphate-buffered saline by the same method. The treatment in adult ducks (180 days old) was similar to that of ducklings. At 1, 2, 3, 5, 7, 9, 12, 15, and 20 days, post-infection the brain tissues were collected for analysis.

### 2.2. Transmission electron microscopy

The brain samples were fixed in 2.5% (v/v) glutaraldehyde in 0.1 M phosphate-buffered saline (PBS, pH 7.4) at 4°C for 24 h. After being washed with PBS, brain blocks were immersed in 1% (w/v) osmium tetroxide for 1 h at 37°C, then rinsed with PBS, dehydrated in ascending concentrations of alcohol (25, 50, 75, 85, 95, and 100%), penetrated with a propylene oxide–Araldite mixture (50% propylene oxide: Araldite), and embedded in Araldite. The ultrathin sections (50 nm) were cut and stained with uranyl acetate and lead citrate, then observed with a transmission electron microscope (Hitachi H-7650, Japan).

## 3. Results

### 3.1. Neurons

**Figure 1:**
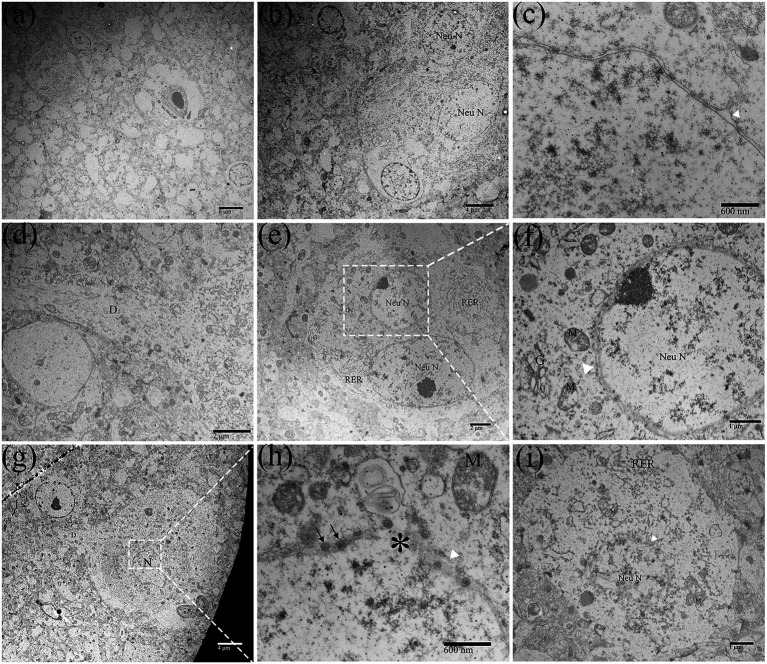
Ultrastructural pathological changes of Duck Tembusu virus (DTMUV)-infected neurons in the duckling brain. **(A)** Brain tissue vacuolization after DTMUV infection. **(B)** Neurons were the main target cells of DTMUV. **(C)** The nuclear membrane gap widened. **(D)** A proximal portion of a dendritic process in an infected neuron. **(E,F)** The nuclear membrane was thickened (white arrowhead). **(G,H)** The nuclear membrane was ruptured (^*^), and residual nuclear pore complexes (black arrow) were visible. **(I)** Nuclear disintegration. RER, rough endoplasmic reticulum; D, dendrite; G, golgi apparatus; Neu N, neuron nucleus; and M, mitochondrion.

**Figure 2:**
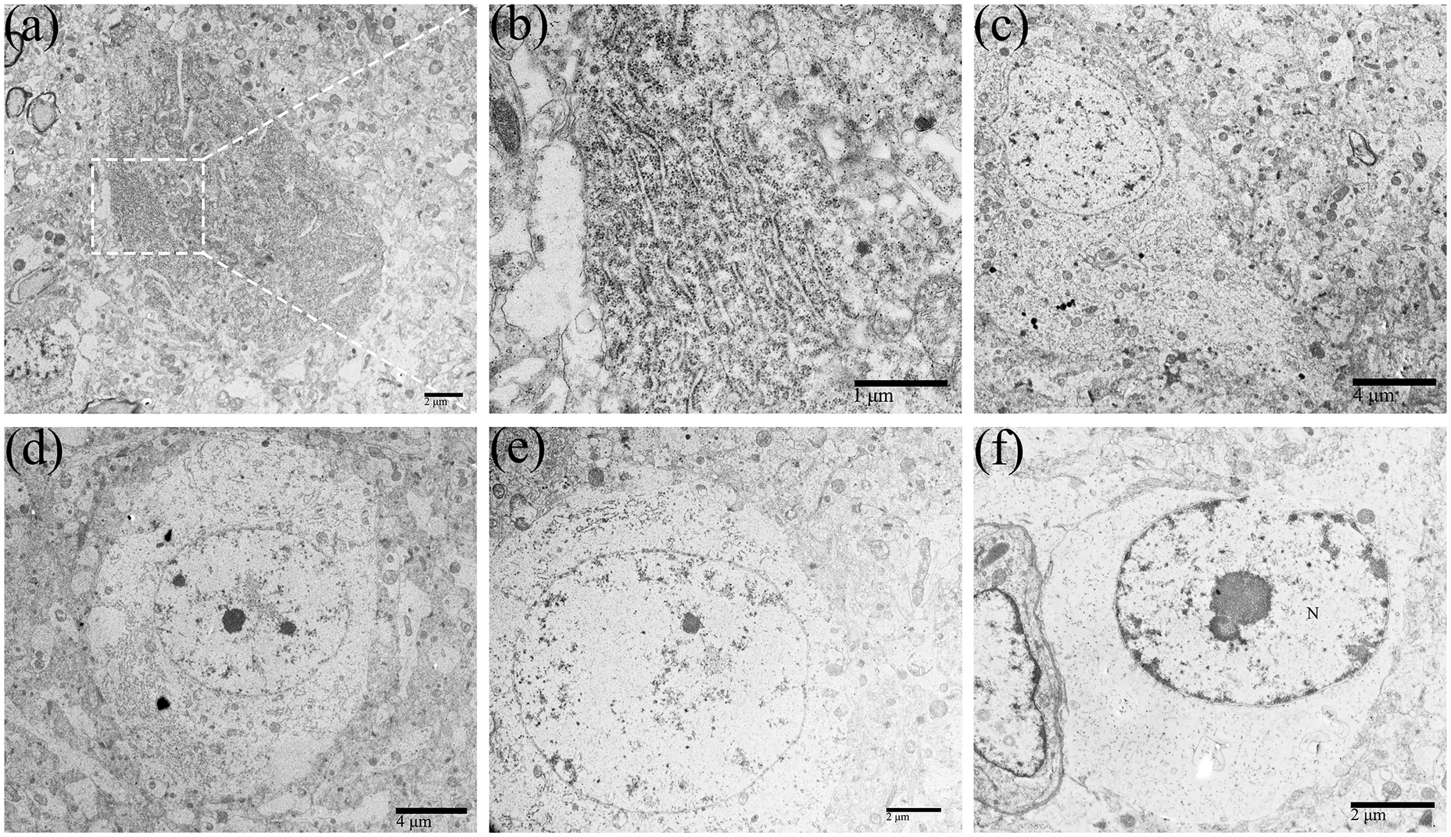
The perikaryon showed degenerative changes in DTMUV-infected neurons in the duckling brain. The organelles in the perikaryon (such as RER, Golgi apparatus, mitochondrion, etc.) were gradually decomposed and disappeared after undergoing morphological changes such as cystic dilation, vacuolation, and fragmentation, leaving the rarefied cytoplasm. **(A)** Normal neurons. **(B)** Lamellar arrangement of RER, also known as Nissl body. **(C–F)** The process of perikaryon degeneration. RER, rough endoplasmic reticulum.

Ultrastructurally, the prominent feature of brain parenchyma in the DTMUV-infected ducklings underwent vacuolation (Figure 1A), and the cytopathic changes seemed to most frequently occur in neurons (Figure 1B). In normal neurons (Figure 2A), there were several ribosomes and rough endoplasmic reticulum (RER) that usually presented regularly arranged lamellar structure (Figure 2B), suggesting that protein synthesis and metabolism in neurons were highly active. DTMUV infection caused extensive damage to neuronal organelles: The RER structures were irregular arrangement and the cisternae of RER presented focal cystic dilatation (Figure 1E); Mitochondria were generally partly damaged, such as swelling and destruction, and mitochondrial cristae were swollen and disappeared (Figures 1F,I); Nuclei were swollen, deformed, and even disintegrated (Figures 1F,I). The nuclear membranes were widened (Figure 1C), thicken (Figure 1F) and ruptured (Figure 1H), and the residual nuclear pore complexes were apparent (Figure 1H). DTMUV-infected neurons also showed extensive organelle lesions in their dendrites (Figure 1D).

**Figure 3:**
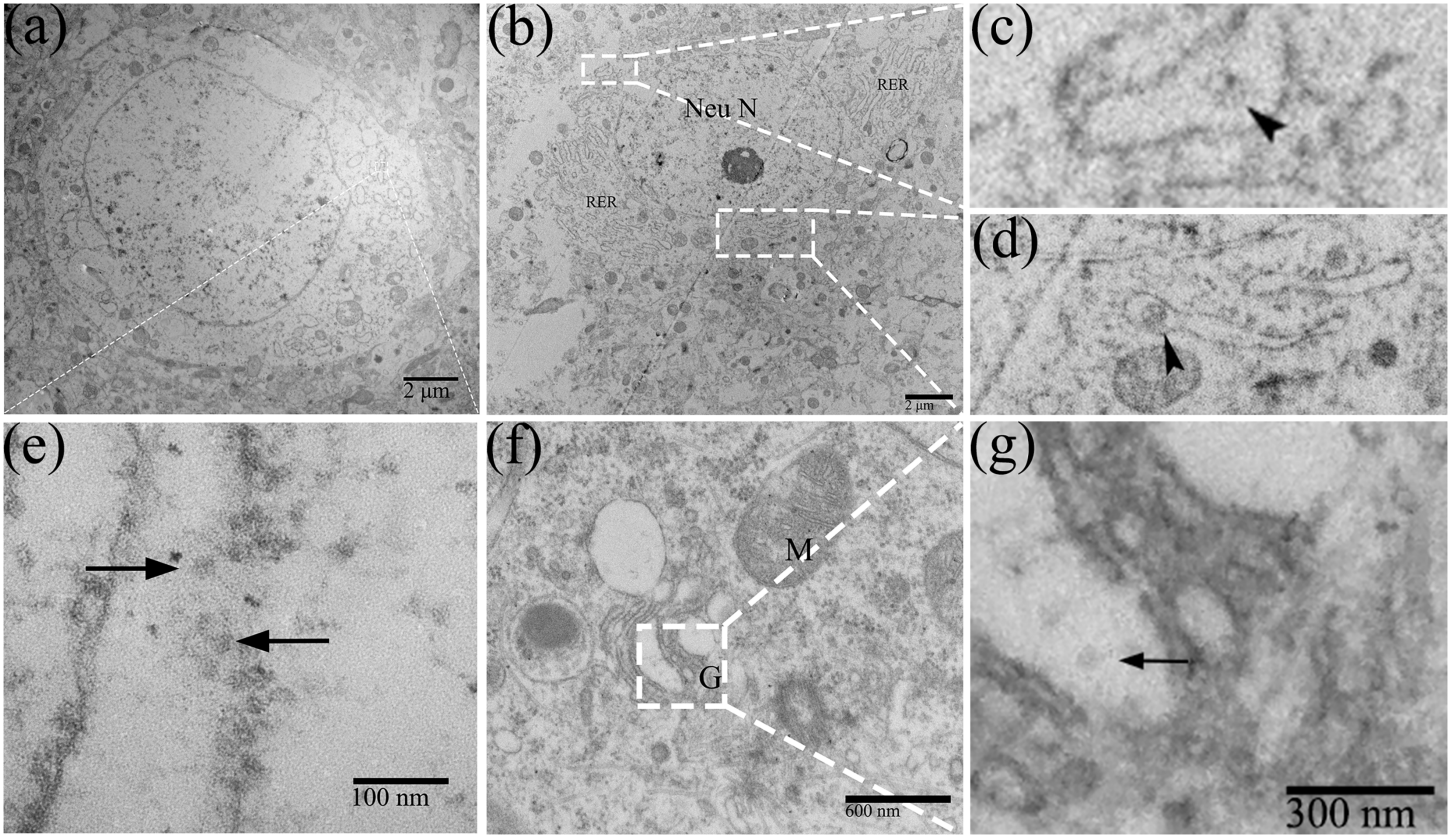
The replication process of DTMUV in neurons in duckling brain. **(A,E)** Dilated RER cisternae contained multiple virions (arrow). **(B–D)** RER cisternae contained membranous vesicles (arrowhead). **(F)** Golgi apparatus showed markedly vacuolar change. **(G)** The Golgi apparatus contains virions (arrow) within its saccules. RER, rough endoplasmic reticulum; M, mitochondrion; G, golgi apparatus; and Neu N, neuron nucleus.

In DTMUV-infected neurons, the cisternae of RER were widened and contained multiple assembling virions (Figures 3A,E). In addition, membranous vesicles of about 100 nm in diameter were frequently been observed in cisternae of RER (Figures 3B–D). The Golgi apparatus in infected neurons also showed markedly vesicular and vacuolar changes (Figure 3F), and virions were found in their swollen saccules (Figure 3G). The distribution of DTMUV virions above reflected their continuous motion in neurons, which were first accumulated in the cisternae of RER and then secreted into the extracellular space *via* the Golgi apparatus secretion channel.

With the replication of DTMUV in the neurons of the duckling brain, the perikaryon of neurons showed degenerative changes (Figures 2C–F). Membranous organelles such as mitochondria, RER, and Golgi apparatus and the electron density were apparently reduced in the perikaryon (Figure 2G). In the early phase of infection, there were rich organelles in the perikaryon, but they showed different degrees of pathology (Figure 2C). In the middle phase, fragmented RER segments were accumulated in partial regions of the perikaryon (Figure 2D), while other regions lacked organelles. In the later phase, the organelles in the perikaryon were decomposed and disappeared (Figures 2E,F).

### 3.2. Neuroglial cell

#### 3.2.1. Astrocyte and oligodendrocyte

**Figure 4:**
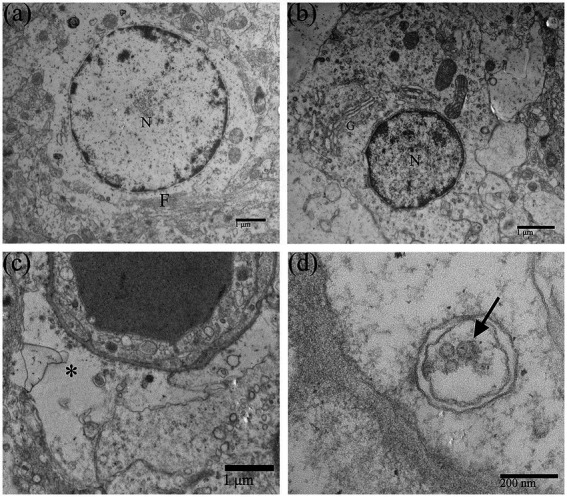
Morphological changes of neuroglial cells in DTMUV-infected duckling brains. **(A)** Astrocyte. **(B)** Oligodendrocyte. **(C)** The astrocytic foot process was swollen, and the glial filaments in the foot process were dissolved and disappeared. **(D)** DTMUV virions (arrow) were encapsulated in vesicles within the astrocytic foot process. G, Golgi apparatus; F, Glial filament. ^*^Astrocytic foot process. N, nucleus.

Duck Tembusu virus infection could also alter the morphological structure of astrocytes (Figure 4A) and oligodendrocytes (Figure 4B), which were less damaged than neurons. After DTMUV infection, the astrocytic foot processes in the duckling brain had obvious pathological changes, such as foot process swelling and dissolution of the glial filaments (Figure 4C). Few assembled virions within coated vesicles in the astrocytic foot processes could be occasionally found (Figure 4D).

#### 3.2.2. Nerve fibers

**Figure 5:**
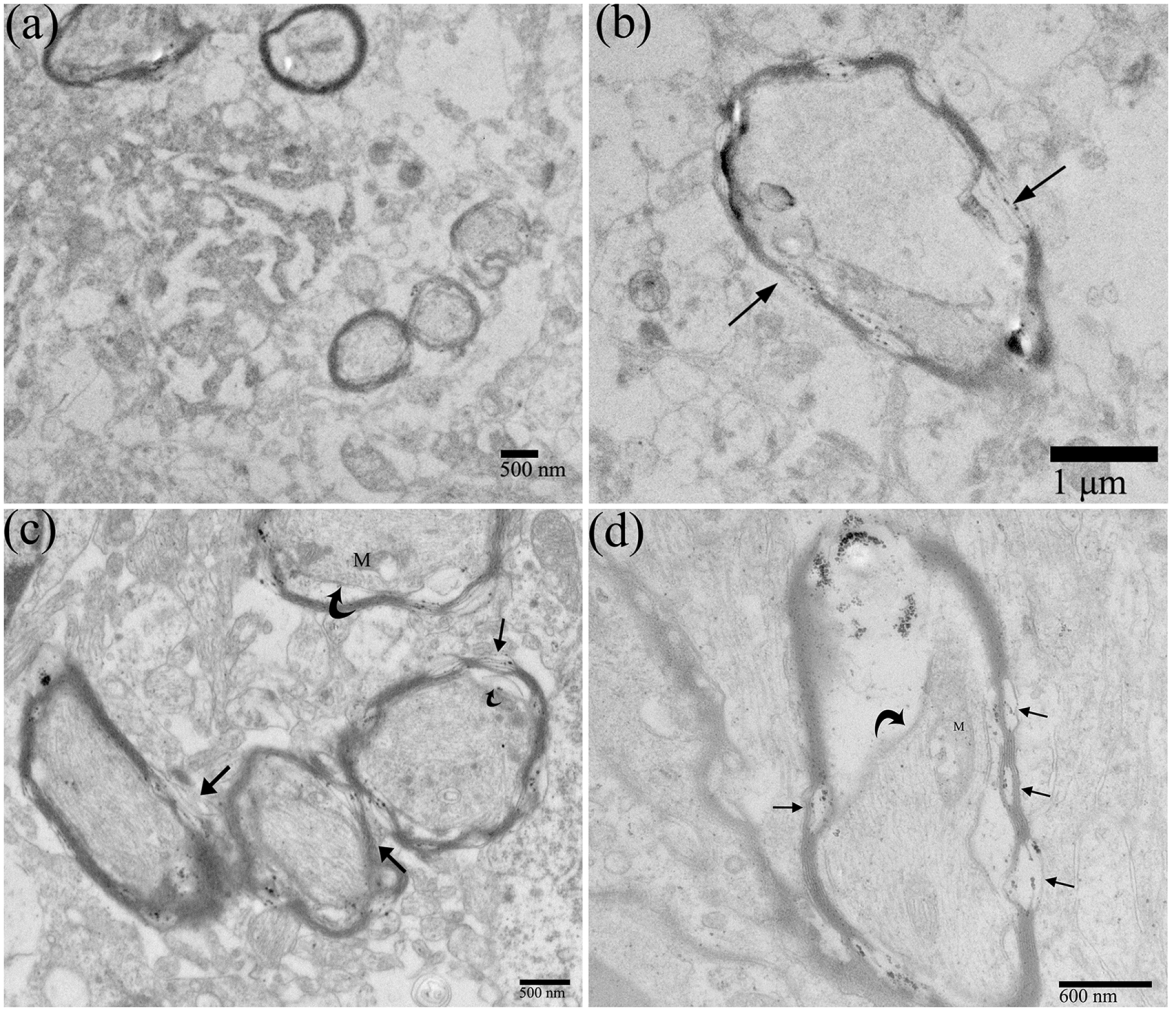
Ultrastructural pathological changes of nerve fibers in DTMUV-infected duckling brain. **(A)** Normal nerve fibers. **(B–D)** The myelin sheaths of myelinated nerve fibers were split and broken (straight arrow), and the inner axonal mesangium was detached (crooked arrow). The mitochondria within the myelinated nerve fibers were damaged. M, mitochondrion.

The myelin sheath of the myelinated nerve fibers of the central nervous system (CNS) is formed by oligodendrocyte protrusions. Normal myelinated nerve fibers’ sheaths were intact and tightly around the axon (Figure 5A). After DTMUV infection, the myelinoclastis and myelinolysis myelinated nerve fibers in the duckling brain were frequently seen. The thickness of the myelin sheath was uneven; The myelin sheath was partially split and relaxed, and its shape became irregular, showing folded and collapsed state (Figures 5B,C). The inner mesangium of the axon of the myelinated nerve fibers was detached and the neurofibrils were dissolved (Figure 5D). The mitochondrial destruction within myelinated nerve fibers was frequently observed.

#### 3.2.3. Microglia

**Figure 6:**
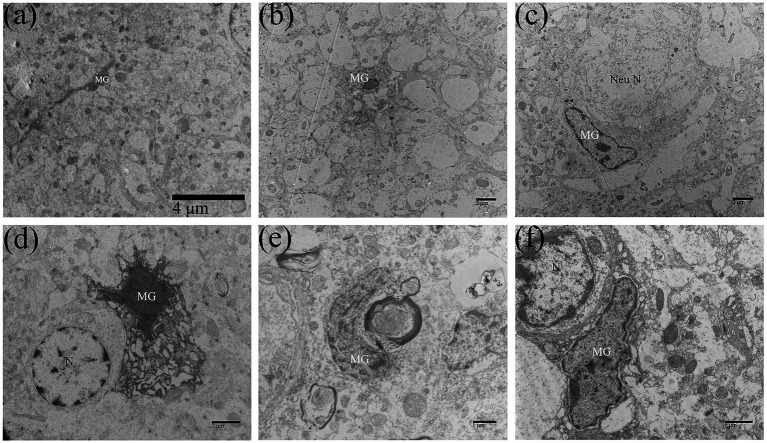
Microglia were activated in DTMUV-infected duckling brains. **(A)** The resting microglia. **(B)** The activated microglia. **(C)** The microglia was phagocytizing the damaged neuron. **(D)** The microglia was phagocytizing the damaged astrocyte. **(E)** The microglia was phagocytizing the damaged myelinated nerve fiber. **(F)** The microglia was phagocytizing the damaged brain microvascular. MG, microglia; Nen N, neuron nucleus; and N, nucleus.

Microglia are immune cells of the CNS. Under normal circumstances, microglia are resting cells, characterized by thin cell bodies, elongated cell processes, highly condensed chromatin in the nucleus, and few organelles in the cytoplasm (Figure 6A). Affected microglia in the brain of ducklings were significantly activated and exerted immune effects. In this study, the activation of microglia usually occurred in the middle and late stages of DTMUV infection (7–15 dpi). As shown in Figure 6B, the primed microglia showed enlarged cell bodies, lower nuclear electron density, shortened cell protrusions, and relatively rich organelles in the cytoplasm. Furthermore, in the DTMUV-infected duckling brain, it could be observed that activated microglia were phagocytosing damaged neurons (Figure 6C), damaged astrocytes (Figure 6D), injured nerve fibers (Figure 6E), and broken microvessels (Figure 6F).

### 3.3. Neuropil

**Figure 7:**
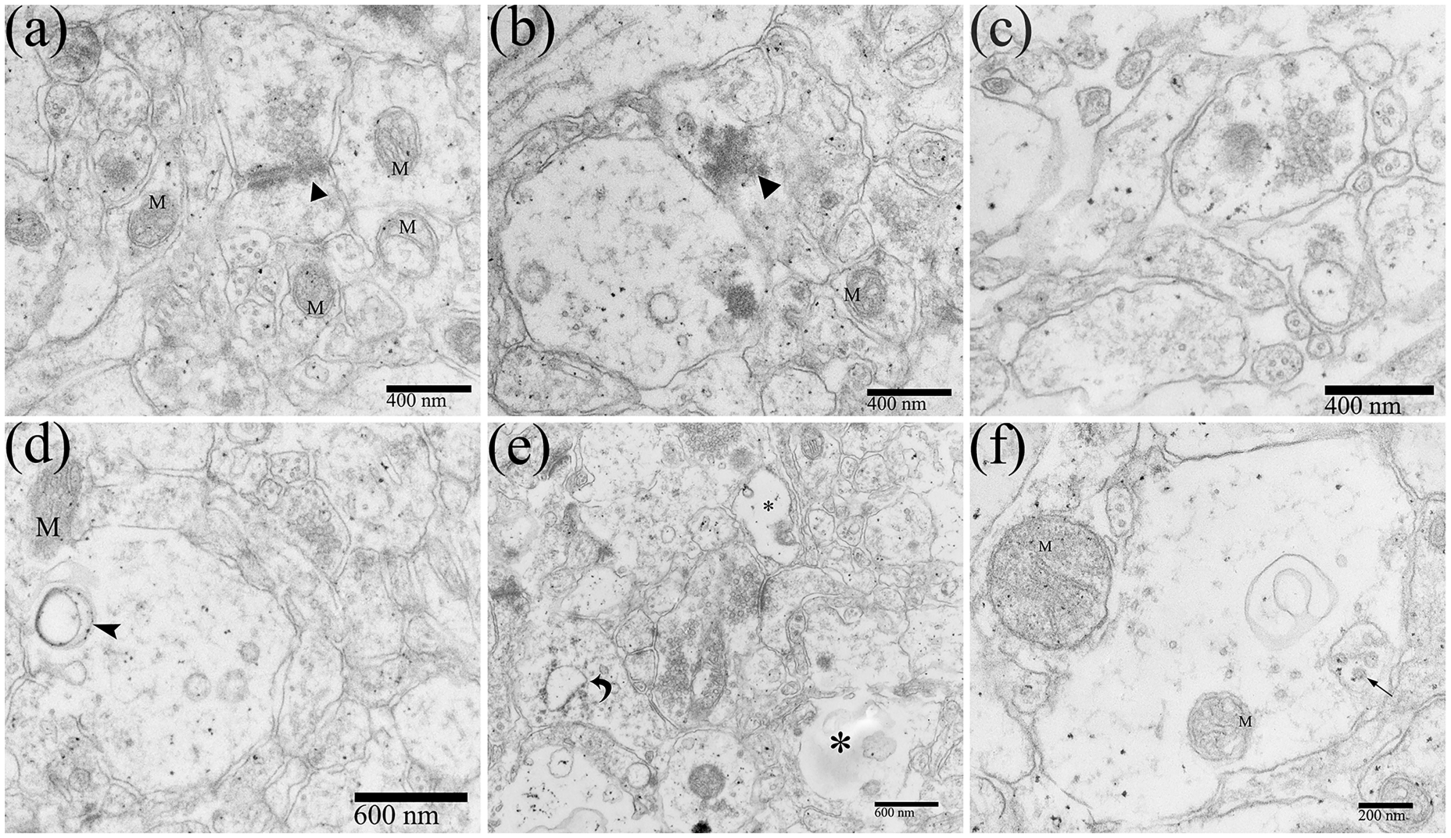
Ultrastructural pathological changes of the neuropil in the DTMUV-infected duckling brain. **(A,B)** Abnormal synapse structure (black triangle). **(C)** The gaps in the components of the neuropil were widened and evacuated. **(D)** The vacuole within dendrite. **(E)** The RER in dendrites was swollen (crooked arrow) and the contents were dissolved (^*^). **(F)** DTMUV virions in vesicle within dendrites. M, mitochondrion.

Neuropil is the area where the dendrites, axons, and glial processes interweave and connect to form a complex network, which is important for information exchange among cells in CNS. Most synaptic connections occur within the neuropil. DTMUV infection could also cause changes in the morphology of neuropil. The synapse structures were abnormal (Figures 7A,B). The spaces between the components of the neuropil became loose and widened (Figure 7C). The organelles in the neuropil were also partly damaged, such as mitochondrial damage and mitochondrial crista swelling (Figures 7A,F), and RER dilating (Figure 7D). Vacuoles and dissolution of dendrite contents occurred in the dendrites of neuropils (Figure 7E). Virions were also observed in the dendrite vesicles (Figure 7F), suggesting that DTMUV could also be assembled in these sites.

### 3.4. Brain microvascular

**Figure 8:**
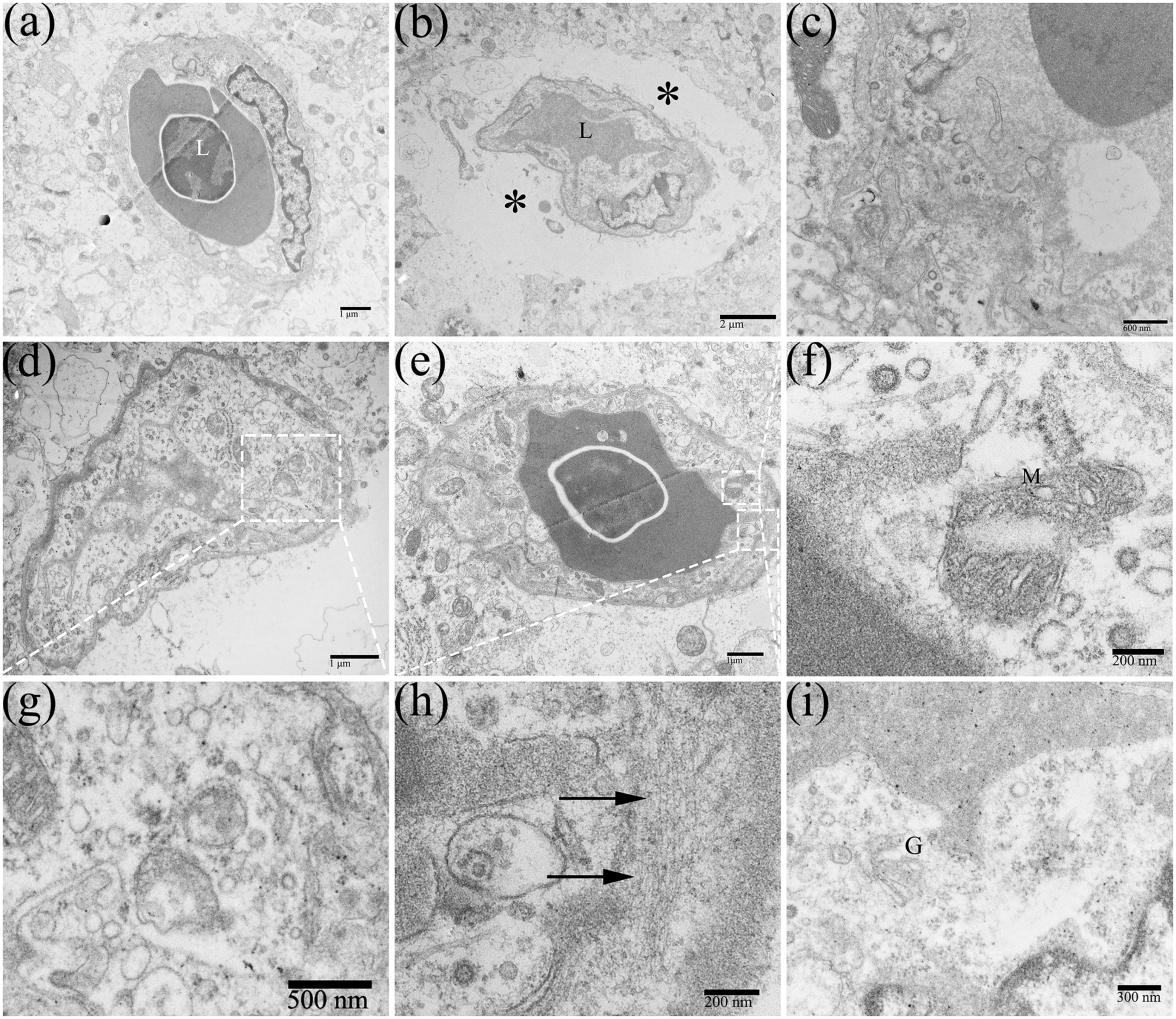
Edema around the capillaries in the duckling brains. **(A)** Normal brain capillary. **(B)** Edema around capillaries (^*^). **(C)** Disorders of the capillary structure. **(D,G)** Pinocytosis vesicles were abundant in capillaries. **(E,F,H,I)** Ultrastructural cytoplasmic changes in brain microvascular endothelial cells (BMECs), such as mitochondrial destruction, increased microfilaments (arrow), and the slightly swollen Golgi apparatus. L, lumen; M, mitochondrion; and G, golgi apparatus.

Cerebral microvessels are continuous capillaries, which together with pericytes and glial cell foot processes constitute the blood-brain barrier (BBB). The special structure restricts the macromolecules and toxic substances in the blood from entering the CNS, to maintain the relative stability of the internal environment of the nerve tissue. Under normal circumstances, the lumen surface of the brain microvascular is smooth, with clear layers and uniform thickness. The basement membrane of the capillaries was clear and complete, and the structure of the connective complex between brain microvascular endothelial cells (BMECs) was clear and dense (Figure 8A). After DTMUV infection, the duckling brain capillaries frequently occurred with visible edema, which was usually observed in the middle and late stages of DTMUV infection (Figure 8B), with low electron density and loose structure in the area around the vessel, and the capillaries wall was disordered and its thickness was uneven (Figure 8C). In the DTMUV-infected duckling brain, the BMECs were swelled, and cytoplasmic organelles were damaged in varying degrees, such as ruptured mitochondria (Figure 8F), the mildly swollen Golgi apparatus (Figure 8I), and increased microfilaments (Figure 8H). Low vesicle rate is one of the characteristics of normal BMECs. However, pinocytotic vesicles and other types of vesicles in BMECs markedly increased after DTMUV infection (Figures 8D,G).

### 3.5. Ultrastructural pathological changes in adult duck brain after DTMUV infection

**Figure 9:**
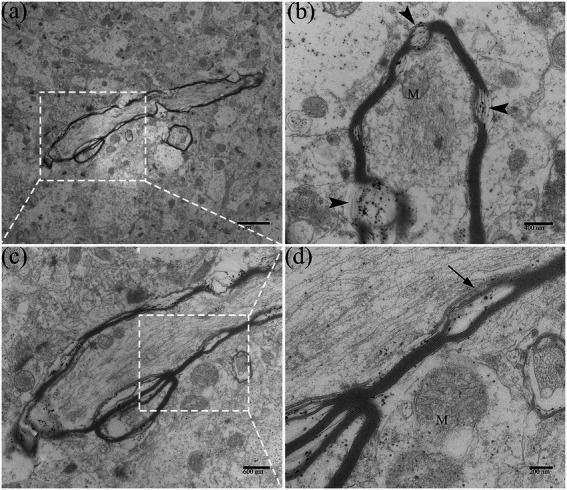
Ultrastructural pathological changes of nerve fibers in DTMUV-infected adult duck brain. **(A)** Myelinated nerve fibers in low-magnification. **(B)** The myelin sheath of myelinated nerve fibers was dissolved (arrowhead). **(C,D)** The myelin sheath of myelinated nerve fibers was split (arrow). Mitochondria within myelinated nerve fibers were also swelled and ruptured. M, mitochondrion.

In this experiment, we observed that the adult ducks did not show apparent neurological symptoms during the DTMUV disease development process. Under electron microscopy, the DTMUV virions were hardly found in adult duck brains. In the DTMUV-infected duck brains, the cellular structure was normal, and no characteristic cytopathological changes were observed, but the myelinated nerve fibers were markedly damaged, such as dissolution of the myelin sheath (Figures 9‒D). Compared with ducklings, the adult duck brain had developed nerve fibers myelin sheath, which was thicker, had more layers, and had higher electron density.

## 4. Discussion

In the present study, we systematically described the pathological changes in the brain, focusing on the relationship between ultrastructural changes of neurons in the brain and virus replication in the neurons after DTMUV infection, which will fill the gap in the understanding of the pathogenesis of DTMUV infection in poultry and provide some guidance for the treatment of DTMUV disease. In addition, DTMUV is a representative of avian flavivirus. So, this study is also of great significance for a comprehensive understanding of the mechanism and treatment of other flavivirus infections.

The replication pattern of flavivirus in host cells has become increasingly clear. Flaviviruses primarily through the clathrin-or caveola-dependent endocytosis pathway reach the host cells. Then, the released genomic RNA of invasive virions is translated into viral proteins, which are assembled in the cisternae of RER, and then, the virions were transported by the Golgi apparatus. In this study, the typical replication process of DTMUV was observed in neurons at the ultrastructural level. DTMUV virions were found in both the RER cisternae and the saccules of the Golgi apparatus. DTMUV infection caused degeneration of neurons, whose morphological changes involved almost all organelles (ER, Golgi complex, mitochondria, nucleus, etc.). Some neurons appeared normal, but there might be virions in whose RER cisternae. Without special attention, these infected neurons might be ignored. Knowing the distribution of the virions in cells could be of great help in finding mildly infected neurons. With the replication of the virus, membranous organelles in the neuron perikaryon gradually decomposed and disappeared, leaving sparse granular matter, which would directly affect the normal biosynthesis and metabolism of neurons, and even threaten their activity. The above results presented the direct interaction between the DTMUV and neurons.

Increased resistance with age is a common feature of many viral infections in the CNS (Johnson et al., 1972; McKendall and Woo, 1987; Ikegami et al., 1997), which may be due to neuronal maturation. Antiviral genes are expressed differently in mature and immature neurons. During the primary culture of neurons, the neurons gradually matured with the culture time, and the expression of interferon (IFN) gradually increased (Farmer et al., 2013). The high expression of immunomodulatory factors in mature neurons can enhance the speed of antiviral response and increase the resistance to viral infections. In addition, many RNA viruses are replicated much more efficiently in immature neurons than in mature ones (Schultz et al., 2015). Therefore, in the present study, infected older ducks may remain normal clinically or develop only transient symptoms.

Recent studies have shown that astrocytes play an important role in chronic neurological dysfunction caused by several neurotropic flaviviruses. The WNV could persist for months in the brains of rhesus monkeys and hamsters (Pogodina et al., 1983; Xiao et al., 2001). Human cases of WNV encephalitis have also been reported that the WNV reactive IgM antibodies remained detectable in the serum of patients who had recovered 18 months or more after infection. Meanwhile, the Tick-borne encephalitis virus (TBEV) could remain latent in the brain tissue of wild rodents for a long period but did not show clinical symptoms of infection (Achazi et al., 2011; Knap et al., 2011; Tonteri et al., 2011). In this study, it was confirmed that DTMUV could infect astrocytes, which indirectly explains the long-term existence of the viruses in the brains of diseased ducks. The WNV (NY385-99 strain) inoculated into mouse primary neurons and astrocytes showed that WNV replicated rapidly and massively in neurons, resulting in neuron destruction and death, whereas WNV replicated slowly in astrocytes and continuously produced infectious viruses (Guzman et al., 2006). The Japanese encephalitis virus (JEV) also had a similar effect to WNV when it infected neurons and neuroglial cells (Chen et al., 2010). The TBEV was able to replicate in astrocytes for 14 days without significant bad effects on cell viability (Potokar et al., 2019). The JEV infection caused obvious morphological changes in astrocyte cell lines *in vitro*, but not in astrocytes in mouse brains *in vivo* (Mishra et al., 2008; Chen et al., 2010). Likewise, in this study, we also found that the pathological changes induced by DTMUV infection in astrocytes were less obvious than those in neurons. The low replication rate of flaviviruses in astrocytes may be the reason why virions are rarely observed *in vivo* experiments, which was also confirmed by our studies.

The major function of oligodendrocytes is to generate myelin sheaths. The integrity of the myelin sheath is critical for maintaining the normal transmission of nerve signals (Moloughney et al., 2018). Demyelination occurs frequently in viral encephalitis, which can lead to CNS dysfunction. Axon regeneration in the CNS is weak because if the myelin sheath is damaged, its damaged part cannot be removed in time (Vargas and Barres, 2007; Brosius Lutz and Barres, 2014). In this study, myelinated nerve fiber injury, such as vesiculataion and fragmentation of myelin sheath (Figures 5, 9), occurred throughout the whole process of the disease. This may be one of the reasons why the ducks developed neurological sequelae after recovery. Fetal myelination was delayed in Zika virus (ZIKV)-infected fetuses, while viral particles were present in oligodendrocytes, and demyelination and axonal damage were also found (Gurung et al., 2019; Rothan et al., 2019). Equally in poultry, in the brains of chickens infected with Marek’s virus, demyelinating plaques were observed as a result of intramedullary fluid accumulation and secondary demyelination (Swayne et al., 1989).

Morphological changes are evidence of microglial activation (Kloss et al., 2001). The resting microglia are branched and perform immunosurveillance functions in the CNS through elongated protrusions. If the brain is attacked and damaged, microglia will retract their protrusions, transform into phagocytic amoeba-like cells, and become the macrophages of the CNS (Orihuela et al., 2016). Microglia activation is normally beneficial to neurons, but when overstimulated, microglia can show neurotoxic aspects. Thus, the duality of microglia function makes their roles in the brain a double-edged sword, which suggests the complexity of microglia effects. The infections of Zika virus (ZIKV) and JEV in flaviviruses were accompanied by the activation of neuroglial cells, especially microglia (Chen et al., 2010; Schultz et al., 2021). Similarly, in our study, DTMUV-induced activated microglia could phagocytose injured neurons (Figure 6C) and glial cells (Figure 6D), etc. And there was a recovery trend in the sick ducks after the alleviation of neurological symptoms, suggesting that the activation of microglia may be involved in the repair of the CNS.

In our previous study, it was confirmed that DTMUV infection induced the disruption of the BBB in ducklings and DTMUV could infect and replicate efficiently in BMECs (Yang et al., 2021). In the present study, we further investigated the ultrastructural cytopathology of the BBB infected by DTMUV. The blood-brain barrier is the biggest obstacle for viruses circulating in the blood to enter the CNS. Astrocytic foot processes are closely associated with BMECs and are susceptible to virions that have crossed the BBB (Potokar et al., 2019). Therefore, astrocytes are considered to be one of the first CNS cells infected by viruses. Astrocytes maintain the barrier function, and their morphological changes affect the permeability of the BBB (Abbott et al., 2006). In this study, DTMUV-mediated morphological changes in the BMECs’ cytoplasm and astrocytic foot processes may be responsible for the changes in the permeability of the BBB. DTMUV infection caused extensive brain perivascular edema (Figure 8B), similar to the effects of JEV and ZIKV in the human brain (German et al., 2006), which is linked with the enhanced permeability of the BBB.

## 5. Conclusion

In summary, the present observations demonstrate that DTMUV infection caused extensive pathological damage to the duckling CNS. DTMUV-induced vacuolated duckling brain, which may be attributed to the reduction of periplasmic organelles of neurons, swelling of astrocytic foot processes, edema around brain microvessels, and dissolution of myelin contents.

## Data availability statement

The original contributions presented in the study are included in the article/supplementary material, further inquiries can be directed to the corresponding author.

## Ethics statement

The animal study was reviewed and approved by Nanjing Agricultural University.

## Author contributions

SY and QC conceived and designed the experiment and revised the manuscript. SY and JW performed the experiment. SY and YS processed the figures. SY prepared and drafted the manuscript. All authors contributed to the article and approved the submitted version.

## Funding

This study was supported by the National Natural Science Foundation of China (grant 31872433).

## Conflict of interest

The authors declare that the research was conducted in the absence of any commercial or financial relationships that could be construed as a potential conflict of interest.

## Publisher’s note

All claims expressed in this article are solely those of the authors and do not necessarily represent those of their affiliated organizations, or those of the publisher, the editors and the reviewers. Any product that may be evaluated in this article, or claim that may be made by its manufacturer, is not guaranteed or endorsed by the publisher.
